# Glycyrrhizin Protects Rats from Sepsis by Blocking HMGB1 Signaling

**DOI:** 10.1155/2017/9719647

**Published:** 2017-04-18

**Authors:** Feng Zhao, Yong Fang, Shuixiang Deng, Xiantao Li, Yun Zhou, Ye Gong, Hechen Zhu, Wei Wang

**Affiliations:** Department of Critical Care Medicine, Huashan Hospital, Fudan University, Shanghai, China

## Abstract

*Background*. HMGB1 acts as an important inflammatory mediator and is a potential therapeutic target for sepsis. Glycyrrhizin (GL), a natural triterpene glycoside derived from licorice, has been demonstrated to inhibit HMGB1 activity. The aim of this study is to explore how GL affects the HMGB1 signaling in sepsis.* Methods*. We used a CLP model of sepsis and in vitro LPS or HMGB1-treated NR8383 cells to examine the effects of GL on expression of HMGB1 and proinflammatory cytokines. Furthermore, we explored the effect of GL on interactions between HMGB1 and RAGE or TLR4 and the activations of NF-*κ*B and MAPKs.* Results*. GL significantly decreased mortality and reduced serum levels of HMGB1 in vivo. GL also attenuated the release and expression of HMGB1 and proinflammatory cytokines. Direct stimulation by HMGB1 elevated the release of proinflammatory cytokines faster than LPS did and it was also inhibited by GL. Furthermore, GL blocked the interaction of HMGB1 with RAGE and TLR4 and suppressed the downstream MAPKs/NF-*κ*B signaling pathway.* Conclusion*. GL may protect rats against sepsis by blocking the interaction of HMGB1 with cell surface receptors and HMGB1-mediated inflammatory responses.

## 1. Introduction

Sepsis is one of the oldest problems in medicine, a systemic inflammatory response syndrome against documented or suspected infection [1]. Because of the increasing number of aging patients and drug-resistant pathogens, the incidence of sepsis has increased to over 750,000 annual cases in the United States, costing nearly $17 billion every year [2]. Sepsis and septic shock are the leading cause of death in intensive care units, with a mortality rate of more than 30% despite advances in critical care medicine [3]. Current clinical therapies for sepsis are limited and include antibiotics, steroidal anti-inflammatory drugs, and early goal-directed therapies (EGDT), which were recommended as guidelines by the International Surviving Sepsis Campaign [4]. Unfortunately, while three large randomized trials found that early administration of antibiotics could increase survival, EGDT did not significantly decrease mortality in septic shock patients compared with the usual care [4]. These findings have prompted an ongoing search for the pathogenesis of sepsis and novel therapeutic methods.

In sepsis, various pathogen-associated molecular patterns (PAMPs) such as lipopolysaccharide (LPS), as well as damage-associated molecular patterns (DAMPs), can stimulate receptors on immune cells like macrophages and monocytes to trigger the release of early proinflammatory mediators (e.g., tumor necrosis factor-*α* (TNF-*α*), interleukin-1*β* (IL-1*β*), and interferon-*γ* (IFN-*γ*)) and the late mediator high mobility group box-1 protein (HMGB1) [5]. The excessive release of these mediators results in countless disturbances of the host immune system accompanied by tissue injury [6, 7]. HMGB1 was first identified as a non-histone DNA binding protein, which was then determined to act as a potential inflammatory cytokine involved in the systemic inflammatory response [8]. HMGB1 is secreted from macrophages/monocytes in response to PAMPs, DAMPs, or proinflammatory cytokines and initiates cellular responses by binding several cell surface receptors, including the receptor for advanced glycation end products (RAGE) and toll-like receptors (TLRs) [9, 10]. The level of serum HMGB1 is significantly increased in patients with severe sepsis [8]. Targeting HMGB1 may be an ideal therapy for sepsis and several studies have shown positive results [11–13].

Glycyrrhizin (GL), also known as glycyrrhizic acid, is a natural triterpene glycoside and the major active component of Gancao (licorice root), widely used as a sweetening and flavoring agent in food. As a traditional herbal medicine, GL has been used clinically as a hepatoprotective drug for more than 20 years in China and Japan [14]. Mollica et al. showed that GL could decrease the concentration of HMGB1 by binding directly to it and therefore inhibit its chemoattractant and mitogenic activities [15]. In a previous study, we used a porcine model of endotoxemia and found that GL could improve systemic hemodynamics, enhance pulmonary oxygen exchange, and protect against damage to multiple organs. These effects were attributed to its regulation of excessive immune responses by reducing the serum level and gene expression of HMGB1 and other proinflammatory cytokines [16]. The results suggested that GL may act as a HMGB1 suppressor to exert its beneficial effects in sepsis. Most recently, GL was reported to prevent sepsis-induced mortality in rats [17], but the mechanism underlying GL regulation of HMGB1 functions in sepsis is still unclear.

In the present study, we established a rat model of cecal ligation and puncture (CLP) and evaluated the effects of GL on mortality and levels of serum proinflammatory cytokines including HMGB1 in vivo. We also observed the effects of GL treatment on secretion and expression of proinflammatory cytokines in vitro using rat alveolar macrophage NR8383 cells stimulated with LPS or HMGB1. Finally, the influence of GL on the interaction between HMGB1 and cell surface receptors and downstream signaling pathways was analyzed.

## 2. Materials and Methods

### 2.1. Experimental Sepsis Model

To evaluate the therapeutic potential of GL, a polymicrobial sepsis model induced by CLP was employed [18]. Briefly, male Sprague-Dawley rats weighing 300–350 g were anesthetized by intraperitoneal injection of sodium pentobarbital (80 mg/kg) and a 1.5 cm midline abdominal incision was made. The cecum was ligated distal to the ileocecal valve without obstructing intestinal continuity. The ligated cecum was then punctured twice with an 18-gauge needle and gently compressed to extrude a small amount of fecal matter. The cecum was returned to the abdominal cavity and the incision was closed. The sham control rats underwent a laparotomy in which the cecum was exposed but not ligated or punctured. All animals received saline solution (3 ml/100 g) subcutaneously for resuscitation immediately after the surgery. This study was approved by the Animal Ethics Committee of Fudan University (Shanghai, China) and performed in accordance with the Guide for the Care and Use of Laboratory Animals of the National Institutes of Health [19].

### 2.2. Drug Administration

For survival rate analysis, rats with CLP were randomly divided into the CLP group (*n* = 10) and CLP + GL group (*n* = 50; rats received intravenous administration of GL 12 h after CLP for three days) (GL was purchased from Sigma-Aldrich, St. Louis, MO, USA, CDS020796), which was subsequently divided into five groups according to the daily GL dosage (1 mg/kg, 2.5 mg/kg, 5 mg/kg, 10 mg/kg, and 20 mg/kg, *n* = 10/group). For cytokine assays, rats were randomly divided into four groups: sham group (*n* = 6; rats underwent sham operations and received sterile distilled water), sham + GL group (*n* = 6; rats underwent sham operations and received 10 mg/kg GL for three days), CLP group (*n* = 6; rats were subjected to CLP and received sterile distilled water beginning 12 h after CLP for three days), and CLP + GL group (*n* = 6; rats received 10 mg/kg GL beginning 12 h after CLP for three days).

### 2.3. Measurement of Cytokine Levels

For in vivo assays, serum samples were collected at 0, 12, 30, and 60 h after CLP. For in vitro assays, culture medium supernatants were collected at 0, 6, 12, and 18 h after LPS or HMGB1 administration. The levels of HMGB1 (LSBio, Seattle, WA, USA), TNF-*α*, IL-1*β*, and IL-6 were determined using enzyme linked immunosorbent assay (ELISA) kits (all from R&D Systems, Minneapolis, MN, USA) according to the manufacturer's instructions.

### 2.4. Cell Culture

Rat alveolar macrophage NR8383 cells were obtained from the American Type Culture Collection (Rockville, MD, USA) and cultured in Ham's F12K medium (Gibco, Waltham, MA, USA) supplemented with 15% FBS and 1% penicillin/streptomycin at 37°C. NR8383 cells were seeded in six-well plates at a density of 1 × 10^5^ cells/well and stimulated by LPS (1 *μ*g/ml, Sigma-Aldrich) or HMGB1 (1 *μ*g/ml, R&D Systems) and incubated with or without GL (Minophagen Pharmaceutical Co., Tokyo, Japan).

### 2.5. Real-Time PCR

Total RNA was extracted from NR8383 cells using Trizol reagent (Invitrogen, Carlsbad, CA, USA) and reverse transcribed using the Superscript First Strand cDNA Synthesis Kit (Invitrogen) according to the manufacturer's instructions. PCR amplification was carried out using a One Step SYBR® PrimeScript™ RT-PCR Kit (Takara, Otsu, Shiga, Japan) on a StepOnePlus real-time PCR system (Applied Biosystems, Foster City, CA, USA). The primers used for PCR are shown in Table 1. The cycling conditions were 5 min initial denaturation at 94°C followed by 40 cycles of denaturation at 94°C for 30 s, annealing at 60°C for 1 min and extension at 72°C for 1 min, and final extension at 72°C for 10 min. The relative levels of cytokine mRNAs were calculated by the 2^−ΔΔCt^ method [20] and normalized to *β*-actin expression as endogenous control.

### 2.6. Coimmunoprecipitation Assay

After various treatments, NR8383 cells were washed with PBS and lysed using 1x RIPA buffer. Lysates were centrifuged at 12,000*g* for 10 min and then incubated overnight at 4°C with 5 *μ*g/ml of either anti-RAGE monoclonal (Santa Cruz, Dallas, TX, USA) or anti-TLR4 monoclonal antibody (Abcam, Cambridge, UK) on protein A agarose beads (Sigma-Aldrich). Immune complexes were then washed with lysis buffer and boiled with SDS sample buffer for 5 min. Finally, the samples were assayed by western blotting.

### 2.7. Western Blotting

After being extracted with lysis buffer, equal amounts of cellular protein were resolved on 6%–12% SDS-PAGE gels and transferred to polyvinylidenedifluoride membranes. After blocking with 5% nonfat dry milk, the membranes were incubated overnight with different primary antibodies specific for HMGB1 (Abcam), RAGE (Santa Cruz), TLR4 (Abcam), p-I*κ*B*α*, I*κ*B*α*, p-JNK, JNK, p-ERK1/2, ERK1/2, p-P38, P38 (all from Cell Signaling Technology, Beverly, MA, USA), and *β*-actin (Abcam). After washing, the membranes were subsequently incubated with the appropriate peroxidase-conjugated secondary antibody (Abcam) for 1 h at room temperature. The blots were visualized using an enhanced chemiluminescence system (Amersham, Arlington Heights, IL, USA).

### 2.8. Statistical Analysis

Survival data were analyzed by the Kaplan-Meier test. Differences among groups were analyzed using one-way analysis of variance, followed by Tukey post hoc test. Data are presented as means ± standard error of the mean (SEM). The *p* values < 0.05 were considered statistically significant.

## 3. Results

### 3.1. GL Protects Rats against Lethal Sepsis

**Figure 1:**
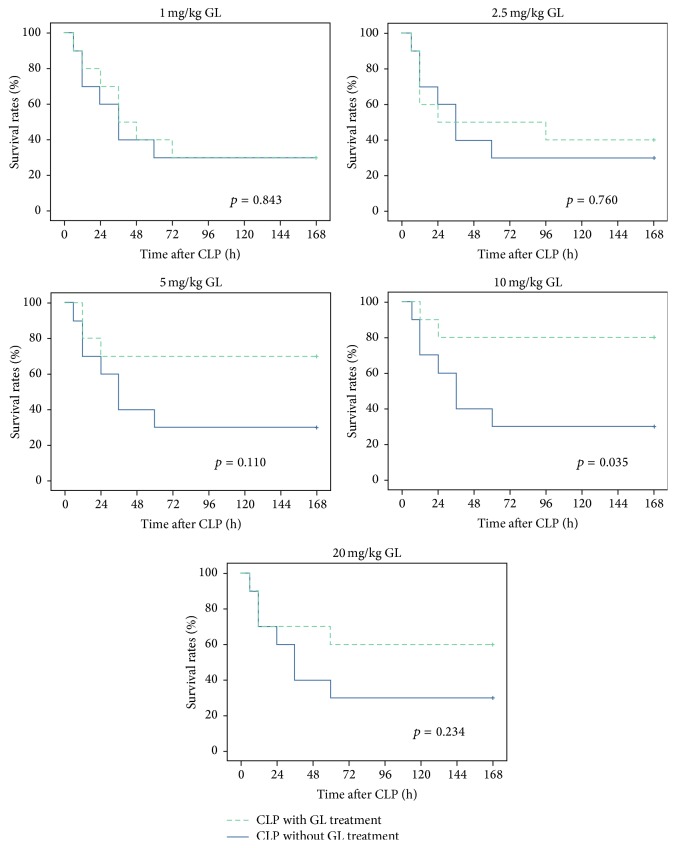
GL treatment reduces sepsis-induced mortality. Rats were given GL at increasing doses for 3 days after the induction of sepsis via CLP. Survival was monitored every 12 h for up to 168 h after CLP. *n* = 10 rats/group. The survival rates of treatment groups were compared with the CLP group.

To evaluate the effect of GL against sepsis, a rat model of polymicrobial sepsis induced by CLP was established. Rats were treated with different doses of GL (1 to 20 mg/kg) for 3 days after CLP and animal survival rates were measured. As shown in Figure 1, sepsis induced by CLP produced 70% mortality in rats within 168 h after CLP, which was improved by increasing doses of GL, resulting in a significantly increased survival rate at 10 mg/kg.

### 3.2. GL Treatment Suppresses the Production of HMGB1 and Proinflammatory Cytokines in Septic Rats

**Figure 2:**
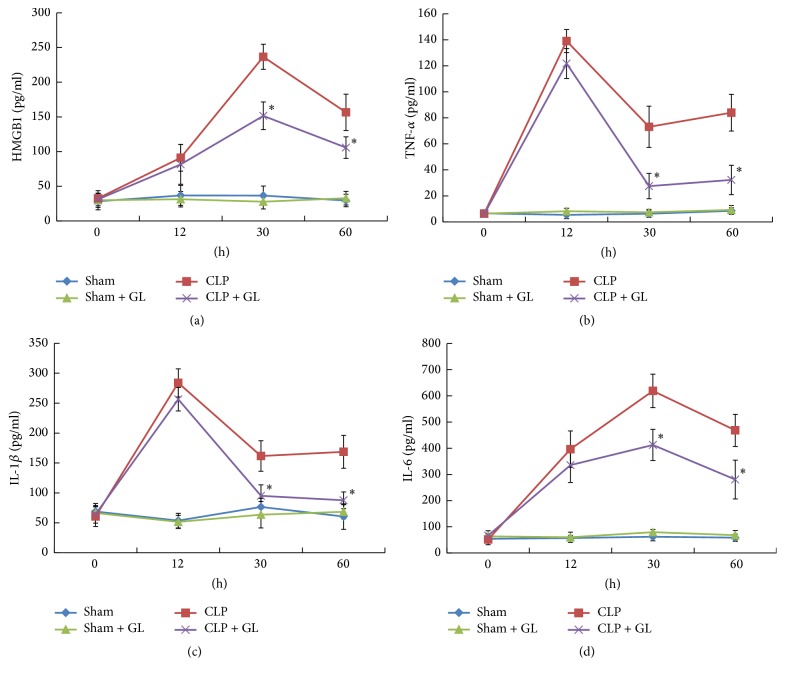
The effects of GL treatment on the production of HMGB1 and proinflammatory cytokines after CLP. Rats received 10 mg/kg of GL for 3 days after CLP or sham surgery. The plasma levels of HMGB1 (a), TNF-*α* (b), IL-1*β* (c), and IL-6 (d) were determined at 0, 12, 30, and 60 h after CLP. Data are presented as means ± SEM. *n* = 6 rats/group. ^*∗*^*p* < 0.05 compared with sham control.

HMGB1 is an important mediator of the inflammatory response which is always exaggerated in sepsis and contributes to the sepsis syndrome [21]. As a potential antagonist of HMGB1 [15, 22], the effect of GL on HMGB1 release and proinflammatory cytokine production in septic rats was evaluated. As shown in Figure 2, plasma levels of TNF-*α*, IL-1, HMGB1, and IL-6 significantly increased after CLP, peaking at 12 h to 30 h. GL treatment did not affect the levels of HMGB1 and proinflammatory cytokines in sham control rats without CLP but did significantly attenuate circulating HMGB1 and proinflammatory cytokine levels in septic rats 30 h and 60 h after CLP.

### 3.3. GL Treatment Attenuated LPS-Induced HMGB1 and Proinflammatory Cytokine Release In Vitro

**Figure 3:**
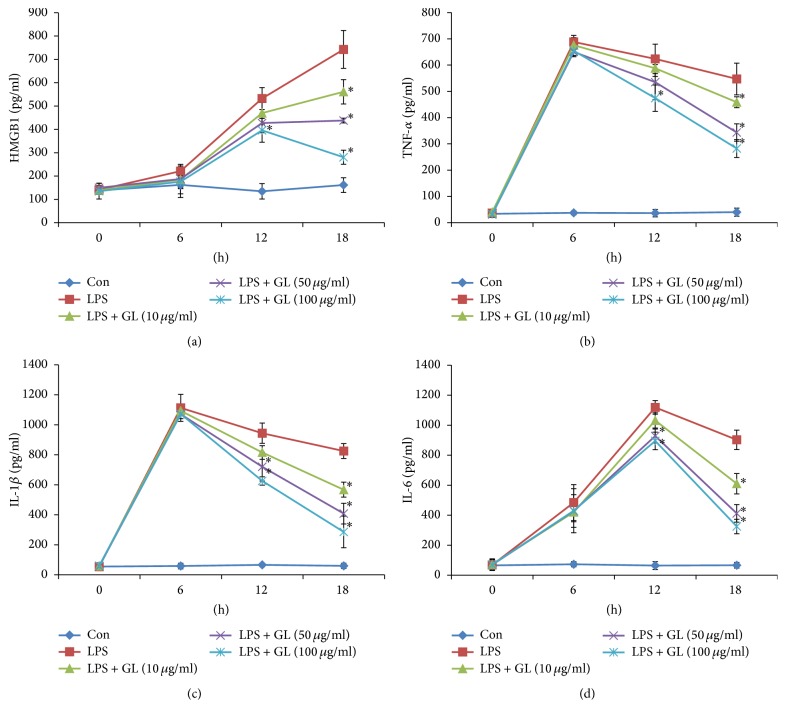
The effect of GL on LPS-induced HMGB1 and proinflammatory cytokine release in vitro. Rat NR8383 alveolar macrophages were stimulated by LPS and treated with or without increasing concentrations of GL. The release of HMGB1 (a), TNF-*α* (b), IL-1*β* (c), and IL-6 (d) was determined by ELISA. Data are presented as means ± SEM. ^*∗*^*p* < 0.05 compared with LPS-treated control.

**Figure 4:**
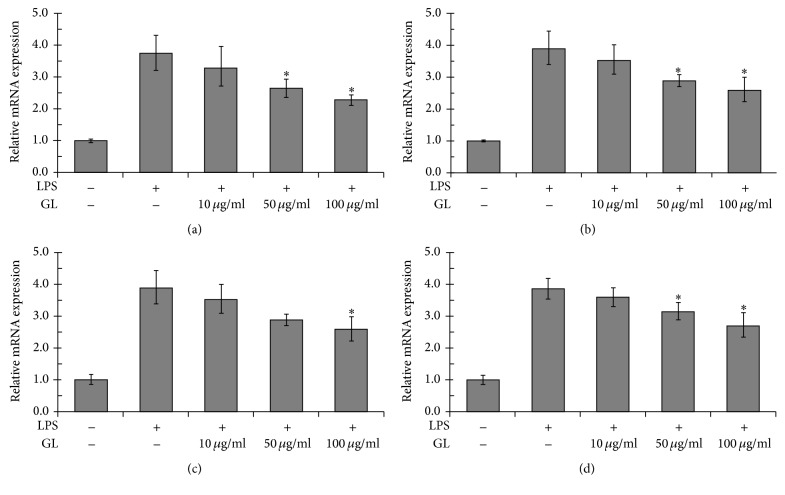
The effect of GL on LPS-induced mRNA expression of HMGB1 and proinflammatory cytokines. Rat NR8383 alveolar macrophages were stimulated by LPS and treated with or without increasing concentrations of GL for 12 h. The mRNA levels of HMGB1 (a), TNF-*α* (b), IL-1*β* (c), and IL-6 (d) were determined by real-time PCR. ^*∗*^*p* < 0.05 compared with LPS-treated control.

We then investigated potential anti-inflammatory effects of GL in vitro using LPS-stimulated rat alveolar macrophages, which are an important producer of HMGB1 and proinflammatory cytokines [23, 24]. The results showed that GL suppressed LPS-induced release of HMGB1, TNF-*α*, IL-1, and IL-6 in a dose-dependent manner after 12 h of LPS treatment (Figure 3). Real-time PCR assay showed that mRNA expression of HMGB1, TNF-*α*, IL-1, and IL-6 in NR8383 cells was also inhibited by GL compared with the LPS-stimulated group (Figure 4).

### 3.4. GL Treatment Attenuated HMGB1-Induced Proinflammatory Cytokine Release In Vitro

**Figure 5:**
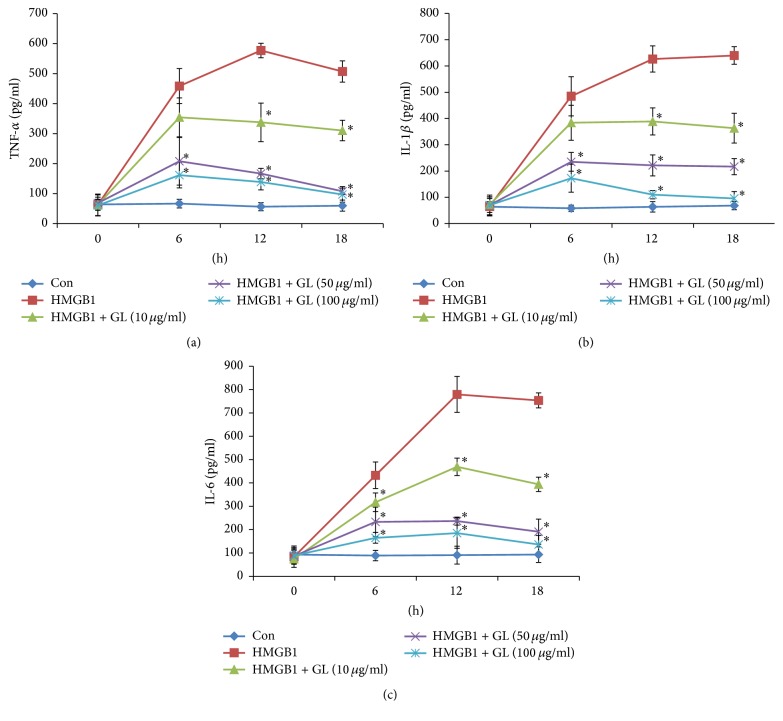
The effect of GL on HMGB1-induced proinflammatory cytokine release in vitro. Rat NR8383 alveolar macrophages were stimulated by HMGB1 and treated with or without increasing concentrations of GL. The release of TNF-*α* (a), IL-1*β* (b), and IL-6 (c) was determined by ELISA. Data are presented as means ± SEM. ^*∗*^*p* < 0.05 compared with LPS-treated control.

**Figure 6:**
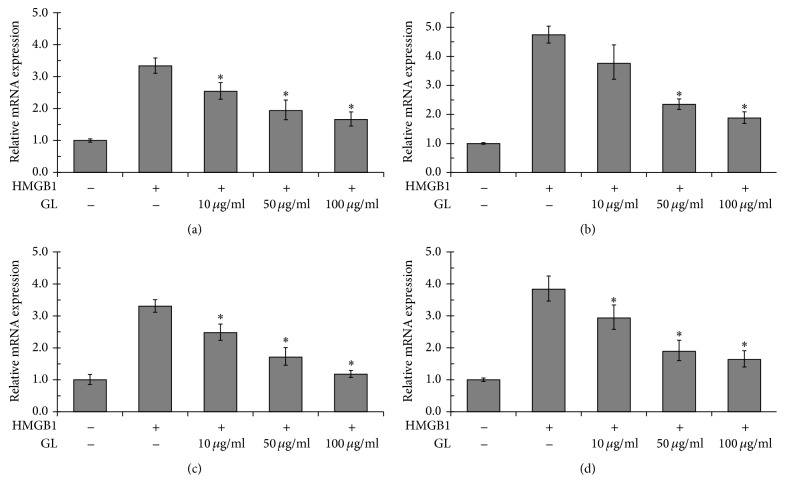
The effect of GL on HMGB1-induced mRNA expression of proinflammatory cytokines. Rat NR8383 alveolar macrophages were stimulated by HMGB1 and treated with or without increasing concentrations of GL for 12 h. The mRNA levels of HMGB1 (a), TNF-*α* (b), IL-1*β* (c), and IL-6 (d) were determined by real-time PCR. ^*∗*^*p* < 0.05 compared with LPS-treated control.

HMGB1 is considered a late mediator of lethal systemic inflammation in sepsis [8]. The results showing that GL did not affect the release of TNF-*α*, IL-1, and IL-6 at 6 h (Figure 3) implied that HMGB1 was the target of GL. To verify that GL blocked the function of HMGB1 in macrophages, the production of proinflammatory cytokines in HMGB1-stimulated macrophages incubated with or without GL was evaluated. As shown in Figure 5, HMGB1 treatment induced the release of TNF-*α*, IL-1, and IL-6 in macrophages. GL at 50 *μ*g/ml and 100 *μ*g/ml significantly reduced the release of proinflammatory cytokines from 6 h to 18 h. Real-time PCR assays showed that HMGB1 could induce mRNA expression of TNF-*α*, IL-1, IL-6, and itself (Figure 6). GL suppressed HMGB1 and proinflammatory cytokine expression in HMGB1-stimulated macrophages in a dose-dependent manner (Figure 6).

### 3.5. GL Inhibits the MAPK/NF-*κ*B Pathway by Blocking Binding of HMGB1 to RAGE and TLR4

**Figure 7:**
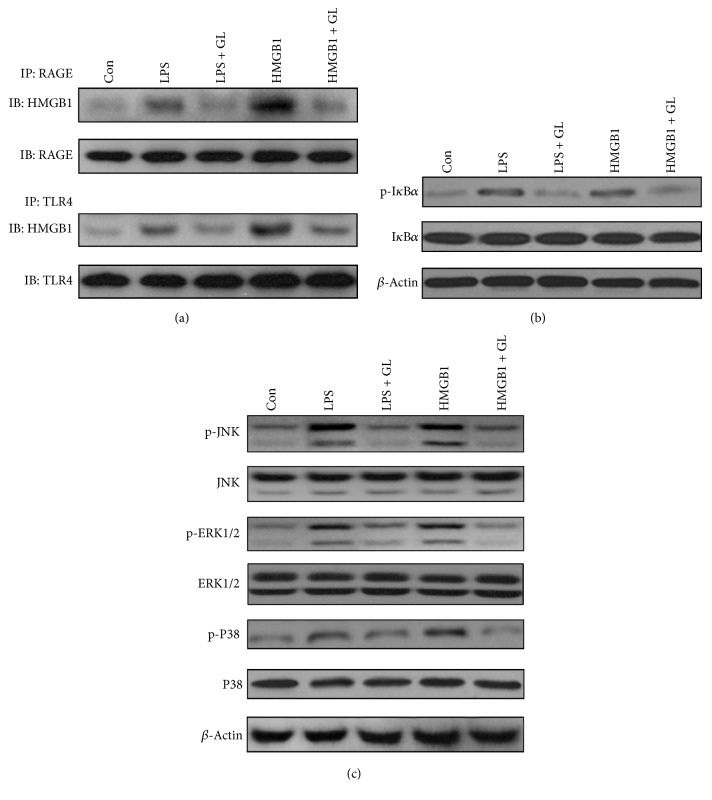
GL inhibits HMGB1-induced NF-*κ*B activation by blocking its binding to RAGE and TLR4. NR8383 cells were stimulated by LPS or HMGB1 and treated with or without increasing concentrations of GL for 12 h. (a) HMGB1-RAGE and HMGB1-TLR4 binding activities were determined by coimmunoprecipitation assays. (b) Activation of I*κ*B*α* was determined by western blotting. (c) Activation of MAPKs including JNK, ERK1/2, and P38 was determined by western blotting.

As a late mediator of sepsis, HMGB1 could modulate the inflammatory cascade through interaction with receptors of various cell types and induce activation of intracellular signaling pathways. RAGE and TLR4 have been identified as the primary receptors for HMGB1 [25]. Activation of these receptors results in the activation of NF-*κ*B, which induces the production of proinflammatory cytokines [26, 27]. To explore the effect of GL on interactions between HMGB1 and RAGE or TLR4, coimmunoprecipitation assays were employed. As shown in Figure 7(a), either LPS or HMGB1 stimulation enhanced the interaction between HMGB1 and RAGE or TLR4, which was notably suppressed by GL incubation. We next analyzed the effect of GL on the activation of NF-*κ*B by measuring the phosphorylation levels of I*κ*B*α*. Higher levels of I*κ*B*α* phosphorylation were detected after LPS or HMGB1 stimulation, indicating NF-*κ*B activation (Figure 7(b)). In the presence of GL, I*κ*B*α* phosphorylation was obviously decreased compared with untreated cells (Figure 7(b)). Furthermore, we determined the influence of GL on mitogen-activated protein kinase (MAPK) activation, including JNK, p38, and ERK, which have all been indicated to link activation of RAGE or TLR4 to phosphorylation and degradation of I*κ*B [27, 28]. We confirmed that LPS and HMGB1 increased the phosphorylation of JNK, p38, and ERK in NR8383 cells. The increased phosphorylation of these MAPKs was appreciably attenuated by GL treatment, suggesting that GL inhibited LPS or HMGB1-induced MAPK activation.

## 4. Discussion

In this study, we found that GL could protect rats against lethal sepsis induced by CLP and suppress the production of HMGB1 and proinflammatory cytokines in septic rats. GL treatment also attenuated the release and expression of HMGB1 and proinflammatory cytokines induced by LPS or HMGB1 in vitro. Furthermore, GL inhibited the MAPK/NF-*κ*B pathway by blocking the binding of HMGB1 with RAGE and TLR4.

Our previous study evaluated the function of GL using an endotoxemia model, whereas a CLP model was used in the present study. This model has been used extensively to investigate the clinical settings of sepsis because it produces polymicrobial septicemia and the full spectrum of sepsis, ranging from acute to chronic pathophysiological processes [29]. In our study, GL treatment at a dose of 10 mg/kg significantly decreased mortality caused by CLP. Interestingly, at the highest dose (20 mg/kg), GL showed reduced efficacy. Recently, Zhao et al. [30] found that 50 mg/kg of glycyrrhizic acid greatly improved the survival rate of rats with sepsis. These differences in the effective dose of GL may be caused by different treatment frequency. The appropriate dose and intervention time for the use of GL in sepsis will require further investigation.

Our previous study and other researches have shown the anti-inflammatory properties of GL in endotoxemia [16] and ischemia-reperfusion injury. Consistently, we found that GL significantly attenuated circulating HMGB1 and proinflammatory cytokine levels in septic rats. To investigate the underlying mechanism, we utilized LPS-stimulated rat alveolar macrophage NR8383 cells, which are a major source of proinflammatory cytokines. The results indicated that GL inhibited LPS-induced release of HMGB1, TNF-*α*, IL-1*β*, and IL-6, which can be attributed to the suppressive effect of GL on cytokine mRNA expression. Notably, the reductions in cytokine levels occurred after GL treatment for 12 h, implying that GL may function as an inhibitor of HMGB1 in LPS-stimulated NR8383 cells. This was further verified by the finding that GL could attenuate the release and expression of proinflammatory cytokines induced by direct HMGB1 stimulation at an early stage. Interestingly, our results showed that HMGB1 mRNA level could be regulated by HMGB1 treatment, implying that there may be a positive feedback and the mechanism is worth further exploration.

HMGB1 has been shown to induce proinflammatory cytokine expression through interaction with cell surface receptors and subsequent activation of intracellular signaling by MAPKs and NF-*κ*B [25]. In the present study, we found that, in addition to direct stimulation of HMGB1, LPS also enhanced the binding of HMGB1 to cell surface receptors TLR4 and RAGE, thereby activating the MAPK/NF-*κ*B signaling pathway. This suggests that HMGB1 mediated the LPS-stimulated inflammatory cascade responses in rat macrophages. GL treatment reduced the interaction of HMGB1 with TLR4 and RAGE, probably by blocking the binding of HMGB1 fragments to TLR4 or RAGE. Schröfelbauer et al. found that the anti-inflammatory effects of GL were specific for membrane-dependent receptor-mediated stimuli [31], and our study provides an explanation for this. In agreement with our results, Okuma et al. also found that GL could suppress HMGB1-RAGE interaction in rats with traumatic brain injury [32]. Recently, GL and GL derivative were shown to inhibit LPS-induced HMGB1 release from RAW264.7 cells [33, 34]; however, our results suggest that GL could also block the interaction of HMGB1 with its receptors.

In conclusion, GL may protect rats against lethal sepsis induced by CLP and suppress the production of proinflammatory cytokines in septic rats and LPS-stimulated rat macrophages. Furthermore, GL inhibited the expression of proinflammatory cytokines by blocking the interaction of HMGB1 with RAGE and TLR4, thereby inhibiting the downstream MAPK/NF-*κ*B pathway.

## Figures and Tables

**Table 1:** The sequences of primers for PCR.

Primer	Sequence	
HMGB1	Sense	5′-ATTGCTGCCTACAGAGCTAAA-3′
	Antisense	5′-GTCGTCTTCCTCTTCCTTCTTT-3′
TNF-*α*	Sense	5′-ACCTTATCTACTCCCAGGTTCT-3′
	Antisense	5′-GGCTGACTTTCTCCTGGTATG-3′
IL-1*β*	Sense	5′-TCCCTGAACTCAACTGTGAAATA-3′
	Antisense	5′-GGCTTGGAAGCAATCCTTAATC-3′
IL-6	Sense	5′-GAAGTTAGAGTCACAGAAGGAGTG-3′
	Antisense	5′-GTTTGCCGAGTAGACCTCATAG-3′
*β*-Actin	Sense	5′-ACAGGATGCAGAAGGAGATTAC-3′
	Antisense	5′-ACAGTGAGGCCAGGATAGA-3′
